# Antiviral Potential and Chemical Composition of Wild *Baccharis crispa* Spreng. Populations (Asteraceae) from Córdoba, Argentina: Perspective on Population Variability

**DOI:** 10.3390/plants13213077

**Published:** 2024-11-01

**Authors:** Giuliana Lingua, Ana Guadalupe Chaves, Juan Javier Aguilar, Florencia Martinez, Tomás Isaac Gomez, Kevin Alen Rucci, Lorena E. Torres, Carmen Ancín-Azpilicueta, Irene Esparza, Nerea Jiménez-Moreno, Marta Contigiani, Susana Nuñez Montoya, Brenda S. Konigheim

**Affiliations:** 1 Instituto de Virología “Dr. J. M. Vanella”-Argentina, Facultad de Ciencias Médicas, Universidad Nacional de Córdoba, Enfermera Gordillo S/N, Ciudad Universitaria, Córdoba X5000HUA, Argentina; giulianalingua@unc.edu.ar (G.L.); juan.javier.aguilar@unc.edu.ar (J.J.A.); florencia.martinez@unc.edu.ar (F.M.); kevin.rucci@mi.unc.edu.ar (K.A.R.); martascontigia@hotmail.com (M.C.); 2 Consejo Nacional de Investigaciones Científicas y Técnicas (CONICET), Godoy Cruz 2290, Cuidad Autónoma de Buenos Aires C1425FQB, Argentina; 3 Cátedra de Genética, Facultad de Ciencias Agropecuarias, Universidad Nacional de Córdoba, Av. Valparaíso S/N Ciudad Universitaria, Córdoba X5000HUA, Argentina; gchaves@agro.unc.edu.ar (A.G.C.); lorenatorres@agro.unc.edu.ar (L.E.T.); 4 Dpto. de Ciencias Farmacéuticas, Facultad de Ciencias Químicas, Universidad Nacional de Córdoba, Edificio de Ciencias 2, Ciudad Universitaria, Córdoba X5000HUA, Argentina; tomasigomez0@gmail.com (T.I.G.); sununez@unc.edu.ar (S.N.M.); 5 Unidad de Investigación y Desarrollo en Tecnología Farmacéutica (UNITEFA-CONICET), Ciudad Universitaria, Córdoba X5000HUA, Argentina; 6 Department of Sciences, Universidad Pública de Navarra, Campus Arrosadía s/n, 31006 Pamplona, Spain; ancin@unavarra.es (C.A.-A.); irene.esparza@unavarra.es (I.E.); nerea.jimenez@unavarra.es (N.J.-M.); 7 Institute for Advanced Materials (INAMAT2), Universidad Pública de Navarra, 31006 Pamplona, Spain

**Keywords:** natural products, population variability, bioactivities, herpes simplex virus, chikungunya virus

## Abstract

Medicinal plants have been explored worldwide as potential alternatives for the prevention and treatment of different diseases, including viral infections. *Baccharis crispa* Spreng. (Asteraceae) is a native medicinal species widely used in South America. Given the influence of genetic and environmental factors on secondary metabolites biosynthesis and accumulation, this study aimed to evaluate the in vitro antiviral activity of four wild populations of *B. crispa* from Córdoba, Argentina, and assess the variability in their bioactivity and chemical composition. The cytotoxicity of chloroform, ethanol, and aqueous extracts from aerial parts was evaluated by the neutral red uptake method. Antiviral and virucidal activity against herpes simplex virus type 1 (HSV-1) and chikungunya virus (CHIKV) were assessed via plaque-forming unit (PFU) reduction assay. Phytochemical analyses of the extracts were conducted using HPLC-ESI- MS/MS. The Puesto Pedernera population showed the strongest antiviral activity, with inhibition rates of 82% for CHIKV and 79% against HSV-1, as well as potent virucidal effects, reducing PFU formation by up to 5 logarithms for both viruses. Remarkably, ethanol extract exhibited the least toxicity and strongest inhibitory activity. Villa del Parque population was inactive. We identified 38 secondary metabolites, predominantly phenolic acids (12) and flavonoids (18), in varying proportions. Delphinidin and delphinidin-3-glucoside are described for the first time in the species. Differences in phytochemical profiles were observed among extract types and populations. Key phenolic compounds showed moderate positive correlations with the evaluated bioactivities, emphasizing the complexity of phytochemical properties and interactions. These results highlight the therapeutic potential of *B. crispa* extracts against viral infections and underscore the importance of considering the geographical source of plant material in bioactivity evaluations.

## 1. Introduction

Infectious viral diseases continue to pose a major threat to global public health, contributing to significant morbidity and mortality worldwide [1]. In recent years, the growing emergence and re-emergence of viral infections represent one of the most worrying aspects of human health [2,3,4]. Among emerging viral pathogens, arthropod-borne viruses stand out due to their ability to spread rapidly in areas with competent mosquito vectors, creating endemic medical problems in developing countries and newly affected regions [5,6]. An example of a re-emerging arbovirus is the chikungunya virus (CHIKV). CHIKV, first identified in Tanzania in 1952 [7], has re-emerged over the last decade, causing large-scale epidemics across Asia, Africa, and the Americas. Its ability to rapidly adapt to new environments and the expansion of its mosquito vectors into new geographic regions, such as Europe and the Americas pose serious public health risks [8,9,10,11]. Notably, in March 2016, the World Health Organization (WHO) notified the first outbreak of chikungunya fever in Argentina, and since then, the arbovirus has been under epidemiological surveillance. So far, no specific treatment has been available to prevent infections by this virus [12].

On the other hand, herpes simplex type 1 virus (HSV-1) is one of three human alphaherpesviruses responsible for widespread infections globally. Since HSV-1 can be transmitted without visible symptoms and leads to lifelong infection, it is crucial to develop interventions, especially for immunocompromised individuals. Although numerous experimental vaccines are being explored, current treatment options are limited to antiviral chemotherapeutic agents (mainly acyclovir and its analogous derivatives) [13]. However, the limited efficacy of these antiviral medications is a significant concern, as they only shorten the recovery period of infection by usually 1–2 days [14]. In addition, viral resistance and side effects of these antiviral therapies pose challenges in the treatment of HSV-1 infection [15].

Despite the approval of nearly 50 synthetic antivirals, many of which are primarily designed for human immunodeficiency virus (HIV) treatment [16], their limited effectiveness leaves several viral infections, such as chikungunya virus (CHIKV), without adequate therapies. Additionally, the emergence of drug-resistant viral strains, such as acyclovir-resistant HSV-1 [17,18], highlights the urgent need for alternative therapeutic approaches [19].

Medicinal plants have been highly valued for millennia as a rich source of therapeutic agents for managing viral disorders. Worldwide, there is an ongoing search for medicinal plants to combat viral diseases due to their accessibility, cost-effectiveness, and lower toxicity compared to synthetic drugs [20]. Plants featuring various secondary metabolites from different chemical classes, such as tannins, terpenoids, alkaloids, and polyphenols, are recognized for their superior antimicrobial properties [21]. This chemical diversity suggests that the strength of the biological activities of a natural product depends on the diversity and quantity of such constituents [19]. Furthermore, it is well established that the content of these active principles depends on the environmental factors that prevail where the plants have developed and on the adaptive response of different genotypes to their environment [22,23,24]. According to the literature, the accumulation of plant secondary metabolites is strongly affected by various environmental factors such as light, temperature, soil water, soil fertility, and salinity. In many cases, a change in a single factor can significantly alter the content of secondary metabolites, even if other conditions remain constant [25,26,27,28,29]. For this reason, the quality of a product of plant origin with therapeutic destination is defined in the first instance by the genotype and, subsequently, by its interaction with the environment [30].

*Baccharis crispa* Spreng. (Asteraceae) is commonly known in Argentina as “carqueja” and is popularly used as an antiseptic, antirheumatic, cholagogue, diuretic, and hepatic agent [31,32]. In addition to these uses, recent studies have shown that several species of the *Baccharis* genus, including *B. crispa*, exhibit antiviral activity. Aqueous and organic extracts of several species of the *Baccharis* genus have demonstrated anti-herpetic activity [33,34,35,36,37,38,39,40]. Hydroalcoholic extracts from *B. crispa* have been reported to inhibit the replication of the vesicular stomatitis virus and poliovirus [41]. These results emphasize the need to continue with this line of research to improve the knowledge and validation of the antiviral properties of this vegetal species.

Therefore, in this work, extracts obtained from four wild populations of *B. crispa* from the province of Córdoba (Argentina) were evaluated in terms of variability in their (i) in vitro bioactivities (cytotoxic and antiviral activity) against herpes simplex type 1 and chikungunya viruses; and (ii) its chemical composition.

## 2. Results

### 2.1. Cytotoxicity Assay

From the analysis of the plots of cell viability (%) vs. concentration of each extract (Supplemental Material, Figure S1), the values of the cytotoxic parameters were estimated. As shown in Table 1, aqueous extracts (Aqu) were less cytotoxic than organic extracts (CHCl_3_ and EtOH), regardless of the population evaluated. Among the four populations evaluated, the EtOH extract of Villa del Parque (VP) and the CHCl_3_ extract of Puesto Pedernera (PP) proved to be the most cytotoxic, with values close to 100 μg/mL. Since some authors point out that CC_50_ values ≤ 20–30 μg/mL should be considered cytotoxic extracts [34], all tested extracts were non-cytotoxic.

Significant differences were found in the cytotoxicity between populations within the type of extract for CC_50_ (DGC, *p* ≤ 0.05). Considering subtoxic concentrations (SubTC), no significant differences were found in the cytotoxic effect of the aqueous extract of VP and PP populations and the CHCl_3_ extracts of Tala Cañada (TC) and PP populations (Table 1).

### 2.2. Plaque Reduction Assay

#### 2.2.1. Antiviral Assays

Those extracts showing an inhibition greater than 50% were selected as a cut-off point. According to this criterion, seven extracts showed antiviral activity against CHIKV and five for HSV-1 (Table 1). The antiviral activity of the extracts was also dose-dependent (Supplementary Material, Figure S2). The inhibition percentage (%I) obtained at the highest concentration evaluated (SubTC) of each extract is shown in Table 1. The population of VP was inactive regardless of the extract and virus evaluated. PP was the wild population with better inhibitory activity on both viruses, being more active against CHIKV than HSV-1 (Table 1). Specifically, the organic extracts (CHCl_3_ and EtOH, *p* > 0.05) showed higher I% than the Aqu extract on CHIKV, and the EtOH extract was the most effective on HSV-1. However, the antiviral activity of the Aqu extract of PP on CHIKV is interesting, considering that it is a preparation commonly used by the population (herbal tea). The San Geronimo (SG) and TC populations showed an intermediate activity on both viruses, between 57 and 67%I. Generally, the EtOH extracts of PP, TC, and SG populations were the most active against HSV-1, whereas CHCl_3_ extracts proved to be the most active against CHIKV (Table 1). Assessment of the variability for antiviral activity showed significant differences between populations within each type of extract tested for both HSV-1 and CHIKV (DGC, *p* ≤ 0.05). The selectivity index (SI) values were generally greater than 2. (Table 1).

#### 2.2.2. Viral Inactivation Assay

**Figure 1:**
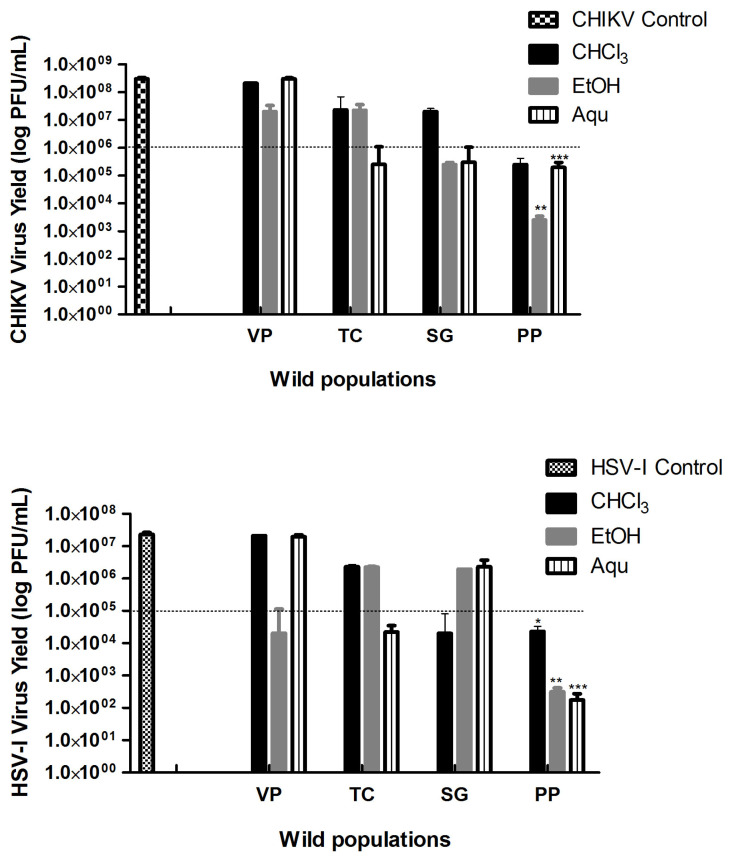
Effect of extracts from four wild populations of *Baccharis crispa* against HSV-1 and CHIKV. Viral inactivation of aqueous (Aqu) and organic extracts (CHCl_3_ and EtOH) from four wild populations of *B. crispa* against HSV-1 and CHIKV. Data represent virus yield (log PFU/mL) compared to viral control (infected and untreated cells), expressed as $\bar{x}$ ± SD (n = 3) of three independent experiments. The dashed line represents the cut-off point for the virucidal activity; extracts below it are positive. * Significant difference between wild populations for the CHCl_3_ extracts *p* ≤ 0.05 (ANOVA DGC test). ** Significant difference between wild populations for the EtOH extracts *p* ≤ 0.05 (ANOVA DGC test). *** Significant difference between wild populations for the Aqu extracts *p* ≤ 0.05 (ANOVA DGC test). CHIKV: chikungunya virus; HSV-1: herpes simplex virus type 1; CHCl_3_: chloroform extract; EtOH: ethanol extract; Aqu: aqueous extract; VP: Villa del Parque population; TC: Tala Cañada population; SG: San Geronimo population; PP: Puesto Pedernera population.

Evaluating the virucidal activity of the extracts against the two viral models showed that all populations had some activity. Only extracts that caused decreases in ≥2 logarithms relative to viral control (VC) were considered with virucidal activity. As shown in Figure 1, the PP population showed the best virucidal capacity in all extracts tested, particularly highlighting the virucidal effect against HSV-1. The EtOH extract of PP was the most active, reducing up to five logarithms in the formation of PFU compared to its control on both viruses. VP, TC, and SG showed intermediate and variable activity depending on the extract. Among the extracts of VP, only the EtOH had activity exclusively against HSV-1; in the case of TC, only the Aqu extract was active for both viral models. In contrast, for SG, only the CHCl_3_ extract influenced HSV-1, whereas the EtOH and Aqu extracts on CHIKV.

Finally, considering the variability in the viral inactivation for the active extracts, the results of HSV-1 showed significant differences between populations for the EtOH and Aqu extracts (DGC, *p* ≤ 0.05). The PP population differs in both cases and has the most significant virucidal activity. In the case of CHIKV, the results obtained were similar since there were also statistical differences between all populations for each extract evaluated (DGC, *p* ≤ 0.05). The PP population is always highlighted since it exhibited the highest inhibition of this virus.

### 2.3. Phytochemical Characterization

The HPLC-ESI-MS/MS analysis of *B. crispa* extracts revealed the presence of 38 compounds, identified by both their retention time (Rt) and their most intense transitions. These compounds are listed in Table S1 of Supplementary Material, together with other phytochemicals that were not identified in any of the samples analyzed. The identified and quantified compounds are distributed in four main categories: phenolic acids, fla vonoids, lignan, and hydroxycoumarin (Supplementary Material, Table S1).

On the other hand, Table S2 of the supplementary material shows the concentration levels of all compounds identified in each extract. As for the phenolic acids, we revealed the presence of 12 of them, including hydroxybenzoic and hydroxycinnamic acids. The main phytoconstituents were caffeic acid, protocatechuic acid, 3-O-caffeoylquinic acid (chlorogenic acid), and 5-O-caffeoylquinic acid (neochlorogenic acid). On the other hand, flavonoids constituted the predominant category of identified compounds in the investigated extracts. The analysis revealed the presence of 18 flavonoids, namely, apigenin-7-glucoside, apigenin, luteolin-7-glucoside, luteolin, genistein, eriodictyol, naringenin, taxifolin, quercetin-3-glucoside, quercetin, rutin, quercitrin, quercitrin-3-glucuronide, quercetin-3-galactoside (hyperoside), kaempferol, isorhamnetin, delphinidin, and delphinidin-3-glucoside. We report the presence of anthocyanins (delphinidin and delphinidin-3-glucoside) in the species for the first time. In addition, we found two lignans in diverse concentrations, pinoresinol, and secoisolariciresinol, regardless of the extract polarity and wild population. Finally, four hydroxycoumarins were identified in the *B. crispa* extracts: esculetin, scopoletin, coumarin, and umbelliferone.

**Figure 2:**
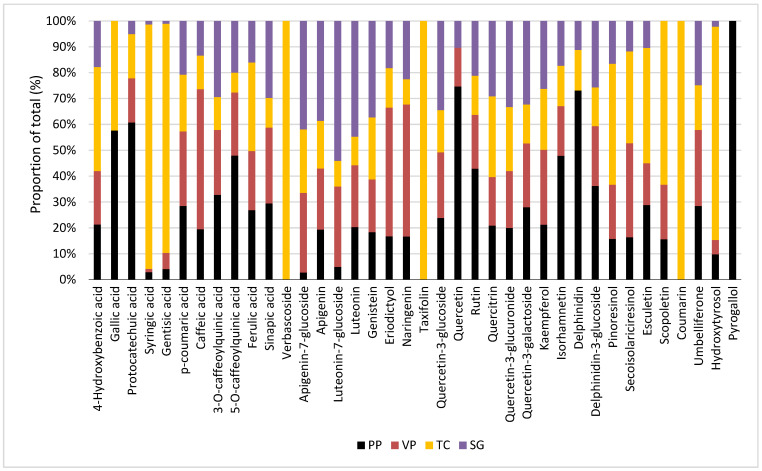
Comparative phenolic profiles of four wild populations of *B. crispa*. Proportion of total (%) of the 38 phenolic compounds found in the wild populations of *B. crispa* (PP, VP, TC, SG). The compounds include phenolic acids, flavonoids, lignan, hydroxycoumarin, and other polyphenols. PP: Puesto Pedernera population (black); VP: Villa del Parque population (red); TC: Tala Cañada population (orange); SG: San Geronimo population (purple).

The chemical profile observed among wild populations was generally similar regarding main constituents but always had different concentrations in each compound identified. As we observed in Figure 2, some compounds were only found in one or two wild populations; for example, pyrogallol is only present in PP; verbascoside (hydroxycinnamic acid derivative), taxifolin (flavanonol), and coumarin (hydroxycoumarin) were only found in TC population, whereas the flavonol, quercetin was not present in these population. Moreover, the anthocyanin delphinidin was not observed in the VP population, and 4-hydroxybenzoic acid and gallic acid were not found in SG and VP.

If we analyze the difference among types of extracts, gallic acid was only present in Aqu extracts of PP and TC populations. Compounds such as gentisic acid, caffeic acid, and derivates flavonols such as quercitrin, quercetin 3-glucuronide, quercetin 3-galactoside, delphinidin 3-glucoside, and umbelliferone were only found in Aqu and EtOH extracts, whereas isoflavone and flavanone, such as genistein and naringenin, were exclusively in organic extracts (Supplementary Material, Table S2).

CHCl_3_ extracts were generally the least diverse and had the lowest concentrations of the identified compounds, regardless of the evaluated wild population. Remarkably, the CHCl_3_ extracts of VP presented the lowest number and concentration of compounds (Supplementary Material, Table S2).

### 2.4. Multivariate Analysis for Phytochemical Profiles and Bioactivities

**Figure 3:**
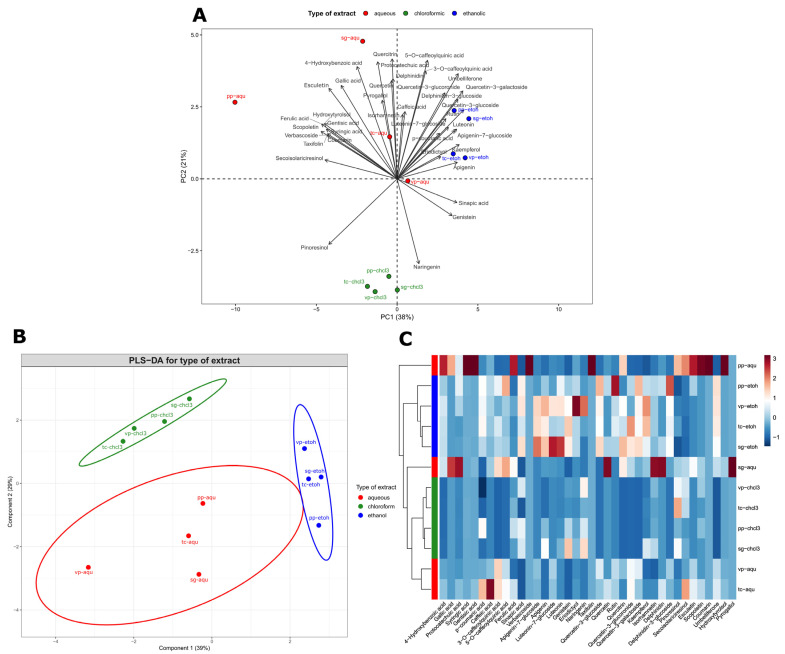
Multivariate analysis for phytochemical profiles and bioactivities of aqueous and organic extracts from four wild populations of *Baccharis crispa*. (**A**) Principal component analysis (PCA) biplot the distribution and abundance of the phenolic compounds. (**B**) Partial least squares discriminant analysis (PLS-DA) illustrating distinct groups between extract types (aqueous, chloroform, ethanol). (**C**) Hierarchical clustering analysis (HCA) displaying the relative abundance of phenolic compounds across different extract types and wild populations, with hierarchical clustering indicating the similarity between samples. The color scale on the right represents normalized values, ranging from −1 (blue, lower concentrations) to +3 (red, higher concentrations).

To visualize the distribution and abundance of phenolic compounds from *B. crispa* across different extracts, a principal component analysis (PCA) was performed (Figure 3A). The first two principal components accounted for 59% of the total variance observed (PC1 = 38%, PC2 = 21%). The first component (PC1) was strongly correlated with a diverse group of compounds, including flavonoids, lignans, and coumarins. Importantly, all the key compounds correlated with PC1 (with correlations greater than 0.75) showed negative correlations. These compounds included syringic acid, gentisic acid, ferulic acid, verbascoside, taxifolin, secoisolariciresinol, esculetin, scopoletin, and hydroxytyrosol. In contrast, the second component (PC2) was associated with a less diverse group of phenolic acids and flavonoids, and all the main compounds correlated with PC2 (r > 0.75) exhibited positive correlations. These compounds were 4-hydroxybenzoic acid, protocatechuic acid, 5-O-caffeoylquinic acid, and quercitrin. The biplot in Figure 3A illustrates the relationships between the compounds and the extracts, revealing the formation of three distinct groups with different phytochemical profiles. Populations are generally clustered according to the extraction solvent (aqueous, chloroform, or ethanol). However, differences in phytochemical profiles were also observed among populations within the same extract type, particularly the aqueous extracts, which displayed the greatest diversity. A notable observation is the clustering of the chloroform extracts, which were grouped together due to their relatively low concentrations of the tested phytochemical compounds.

Hierarchical clustering analysis (HCA) and partial least squares discriminant analysis (PLS-DA) were conducted to explore groupings among the extracts based on their phytochemical composition (Figure 3B,C). Consistent with the PCA results, both analyses (HCA and PLS-DA) revealed the formation of three distinct groups corresponding to the type of solvent: aqueous, chloroform, and ethanol. The extracts from the four *B. crispa* populations (PP, VC, SG, and TC) exhibited variability in their chemical composition, as indicated by their different positions in the dendrogram and PLS-DA plots (Figure 3B,C). In particular, the aqueous extracts showed the highest diversity, with the PP-Aqu extract standing out due to its high concentration of several compounds (e.g., syringic acid, gentisic acid, verbascoside, coumarin, and umbelliferone), setting it apart from the other extracts. On the other hand, some aqueous extracts, such as SG-Aqu, appeared more like chloroform extracts (Figure 3C).

**Figure 4:**
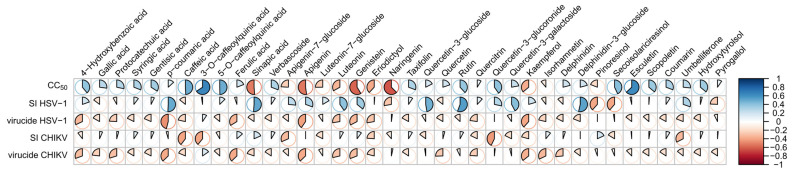
Correlogram showing the Pearson correlation between phenolic compound concentrations of each extract and bioactivities of *Baccharis crispa.* The color gradient represents correlation values, ranging from −1 (red, strong negative correlation) to +1 (blue, strong positive correlation), as shown by the scale on the right. The size and direction of the pie slices within the circles indicate the magnitude of the correlations.

Additionally, a set of Pearson correlations was performed to assess the potentially anti-viral and virucide properties of the measured compounds (Figure 4). For cytotoxicity, compounds like caffeoylquinic acids and esculetin exhibit a higher positive correlation (lower cytotoxicity), as indicated by the blue coloration in the CC_50_ row. Other compounds, such as genistein and naringenin (red color), show higher cytotoxicity. For antiviral activity, we evaluated the correlation between the SI values and the concentrations of the compounds. We expected to find a positive correlation with some compound concentration to consider it a putative antiviral compound; however, most correlations were negative. The compounds with relatively highly positive correlations (and antiviral activity) for HSV-1 were *p*-coumaric acid, sinapic acid, quercetin-3-glucoside, rutin, and delphinidin-3-glucoside. For CHIKV, all correlations were negative, indicating no antiviral activity. For virucidal activity, we expected to find a negative correlation. For HSV-1, relatively correlated compounds were *p*-coumaric acid, apigenin, and kaempferol, whereas for CHIKV, the compounds *p*-coumaric acid, ferulic acid, apigenin, and kaempferol showed negative correlations with virucide activity.

## 3. Discussion

Although there are some reports about the antiviral activity of varied extracts from several *Baccharis* species on a wide variety of viruses: Junín virus, HIV, HSV-1, vesicular stomatitis virus (VSV), poliovirus (PV) and Equid herpesvirus 1 [38,39,40,41,42,43,44], information on *B. crispa* is scarce. Until now, only hydroalcoholic extracts from *B. crispa* have been reported to inhibit the replication of VSV and PV [35]. This activity is probably due to the presence of apigenin (flavonoid with broad-spectrum antiviral activity) with reported antiviral activity against PV in other species of the genus [45,46].

Our work focuses on *B. crispa*, a species native to Argentina whose medicinal and industrial use, mainly in the production of bitter beverages and nutraceuticals, has led to the decline of their wild populations that threatens the conservation of this plant species in some areas of the country. According to the Conservation Priority Index (CPI) for medicinal plants, *B. crispa* is in third place in order of importance as a priority for conservation in the Paravachasca and Calamuchita Valley of the province of Córdoba (Argentina) [40]. In this context, it is crucial to identify wild populations with medicinal interest and prioritize their conservation. External factors or variables (light, temperature, soil water, soil fertility, and salinity) can significantly influence the growth and development of plants, influencing their synthesis of secondary metabolites and thereby altering their phytochemical profiles [25,26,27,28,29]. These environmental factors are crucial for the production of bioactive substances. Considering the critical role of viral infections in human health, we have focused on evaluating the antiviral activity of four wild populations of this plant species.

The results obtained in this work show a significant in vitro virucidal and antiviral effect for all the extracts of the PP population against HSV-1 and CHIKV (Table 1 and Figure 1). In contrast, the rest of the populations showed an intermediate or low antiviral and virucidal activity. The results show that the PP population has a significant virucidal activity, especially on HSV-1, which is reduced by up to five logarithms and has an excellent antiviral activity (51 to 79%I, depending on extract polarity). We have shown that *B. crispa* stands out as another species belonging to the *Baccharis* genus with in vitro antiherpetic activity, and it has also demonstrated its high inhibitory effect on CHIKV (around 74 to 81%I, depending on the polarity of the extract), a pathogenic virus with no therapeutic treatment. These are essential results since they are new to this species and contribute to the knowledge of the bioactivities of the genus. They also reinforce previous data obtained about other *Baccharis* species with antiviral activity on HSV-1 [37].

We found that EtOH and CHCl_3_ extracts from *B. crispa* had better antiviral activity than Aqu extracts (decoction), regardless of the evaluated virus. It is even observed that EtOH extracts were the most active on HSV-1, whereas CHCl_3_ extracts exhibited better action on CHIKV, regardless of the *B. crispa* population tested, except for the VP population, which was inactive for the two viruses assayed.

Considering the SI definition, it can be inferred that when the SI value is high, the cytotoxicity is low, and the effect against the virus is high. As plant extracts are a complex mixture of chemical compounds, each contributing differently to this matrix’s biological effect, their SI values are usually not high [47]. No value for this index defines whether a sample is an excellent antiviral agent; however, some authors consider that an SI ≥ 2 in natural products (extracts or compounds) would indicate a potential antiviral agent [48]. In our study, the SI found in most cases was superior to 2; considering this SI, it can be concluded that there is variability in the bioactivity among the wild populations evaluated, which is more accentuated between VP and PP populations (Table 1). The PP population was the most active in its antiviral and virucidal activity on both viruses, highlighting the EtOH extract as less toxic and with good inhibitory activity against both viral models. Then, with an intermediate activity, SG, followed by TC. The VP population, on the other hand, was discarded as it was inactive regardless of extract type or the virus tested.

Although the chemical profiles observed among wild populations were similar, the population with the best activity (PP) had the highest concentration of identified phenolic compounds. These differences are accentuated when comparing populations with extreme activities (VP and PP) and between de Aqu extracts. We infer that the differences found in biological activities (cytotoxicity and antiviral) among wild populations of *B. crispa* could be related to the variations in the chemical composition obtained. The variation may also reflect selective pressures of ecological and geographical environments (ecotypes) [49,50,51].

We have identified caffeoylquinic acid derivatives (quinic acid with various esterifications with caffeic acid) as the main compounds in the Aqu extracts of *B. crispa*, as observed by Simões-Pires et al. [52]. Also, four hydroxycoumarins were identified in the Aqu and EtOH extracts; in recent years, there have only been a few reports on the coumarin composition of the *Baccharis* genus, and these reports concern *B. grisebachii* Hieron, *B. tricuneata* (L.f.) Pers., and *B. dracunculifolia* D.C. [32,53].

On the other hand, in organic extracts, derivatives of flavonoids and neoclerodane diterpenes were the main isolated compounds [54]. Flavonoids such as genkwanin and apigenin were found mainly in EtOH extracts of aerial parts from *B. crispa* [55]. These flavonoids have shown in vitro antiviral activity against CHIKV, HSV-1, Hepatitis B, African Swine fever, Buffalopox, and Enterovirus-71 [56,57,58]. In our work, the most identified flavonoids were apigenin and luteolin. Although we confirm apigenin as the major flavonoid in the organic extracts evaluated and the literature describes it as a potential antiviral agent, our study showed higher concentrations for this compound in VP extracts, which has no activity against the viruses tested.

We report, for the first time, the presence of anthocyanins (delphinidin and delphinidin-3-glucoside) in the species. This flavonoid has been previously described with antiviral activity against Hepatitis C, West Nile, Zika, and Dengue viruses, all members of the *Flaviviridae* family [59,60].

In our study, correlations were explored to identify relationships between phenolic compounds and biological activities, with a focus on highlighting key compounds with potential antiviral or virucidal properties against HSV-1 and CHIKV and their cytotoxic profiles. Compounds like *p*-coumaric acid, sinapic acid, quercetin-3-glucoside, rutin, and delphinidin-3-glucoside exhibited moderate positive correlations with antiviral activity against HSV-1, as was evidenced by higher SI values (Figure 4). However, for CHIKV, no significant correlations were found. These findings emphasize the complexity of plant-based extracts and the difficulty in identifying the key antiviral constituents within a mixture of compounds. Consequently, the antiviral capacity of an extract might depend not only on the concentration of its antiviral compounds but also on their interactions with other components. Such interactions may include synergistic and/or antagonistic effects, the formation of stable intermolecular complexes, or irreversible reactions between the different components of the extract [61,62]. The moderate correlations observed between a few phenolic compounds and HSV-1 antiviral activity suggest that these compounds could serve as important leads for further investigation. Future work should aim to characterize the interactions between compounds to optimize the antiviral efficacy while minimizing cytotoxicity. Additionally, exploring combination therapies that leverage the synergistic effects of multiple compounds could prove to be a promising strategy for enhancing antiviral potency while reducing potential side effects.

The variability observed among wild populations highlights the importance of considering the origin of the plant material used to carry out bioactivity evaluations. Furthermore, the study of different populations of plant species allows for the selection of the population with bioactivity for future domestication. Another study by our group found that different *Tagetes minuta* L. (Asteraceae) populations have different chemical compositions and antiviral activities [63]. Sartor et al. [64] agree that modifications in environmental conditions (biotic and abiotic), where different individuals of the same species grow, produce variations in the plant’s chemical composition.

## 4. Conclusions

This study lays the basis for continuing the exhaustive study on the antiviral properties of the species *B. crispa*, deepens the background of bioactivity already reported on the genus and species, providing unpublished data on its antiviral activity, being the first record about bioactivities of wild populations of *B. crispa* from the province of Córdoba (Argentina). Moreover, the results obtained contribute to the knowledge of the species to promote its conservation and sustainable use.

## 5. Materials and Methods

### 5.1. Plant Material

Vegetal material (aerial parts) of *Baccharis crispa* Spreng. (Asteraceae) was collected in April of 2014 from four wild populations that grow in different areas of the natural distribution zone of this species in the province of Córdoba-Argentina: Villa del Parque, VP (31°49′14.7″ S, 64°30′17.8″ W, 827 m above sea level); Puesto Pedernera, PP (31°36′21.2″ S, 64°41′14.5″ W, 1515 m above sea level); Tala Cañada, TC (31°21′57.4″ S, 64°57′11.5″ W, 1342 m above sea level) and San Geronimo, SG (31°20′02.3″ S, 64°55′50.7″ W, 1486 m above sea level). The plant material was collected in the same vegetative state, advanced flowering. A voucher specimen of each population was herbarized and deposited in the ACOR herbarium (Facultad de Ciencias Agropecuarias, Universidad Nacional de Córdoba). The specimens were incorporated into the Lucas M. Carbone (LMC) collection. The plant material was air-dried at room temperature, powdered, and kept in light amber containers until processing. 

### 5.2. Preparation of Plant Extracts

#### 5.2.1. Organic Extracts

Forty grams of dried aerial parts from each wild population were initially subjected to maceration with hexane to extract fats and chlorophylls. Subsequently, they were extracted in a Soxhlet extractor with chloroform (CHCl_3_) and ethanol (EtOH). The plant material was in contact with each solvent until depletion. Each extract was concentrated to dryness in a rotary evaporator (RV1 rotary evaporator, Figmay, Córdoba, Argentina) at moderate temperatures (30–40 °C). All organic solvents were previously distilled before use.

#### 5.2.2. Aqueous Extract

Another fraction of dried and ground aerial parts (40 g) was extracted with enough water to cover the plant material at boiling temperature for one hour (decoction). Each aqueous extract (Aqu) was lyophilized (Labconco LYPH LOCK 6, Lyophilizer, Kansas City, MO, USA).

### 5.3. Phytochemical Analysis

The analysis of phytochemicals of extracts from aerial parts of *B. crispa* was performed on an HPLC-ESI- MS/MS system (Sciex, Framingham, MA, USA), which consists of an Exion AC HPLC coupled to a QTRAP 4500 with an electrospray ionization source (ESI). Data were acquired using scheduled multiple reaction monitoring (sMRM). Chromatographic separation was carried out on a Kinetex Biphenyl column (5 µm, 2.1 × 150 mm, Phenomenex, Torrance, CA, USA), attached to a security guard column (Phenomenex, Torrance, CA, USA). The mobile phases comprised 0.1% formic acid in water (solvent A) and 0.1% formic acid in methanol (solvent B). The flow rate was 0.3 mL/min, and the following solvent gradient was used (t in minutes, %B): (0, 5%); (2, 5%); (8, 40%); (16, 50%); (24, 55%); (26, 100%); (28, 100%); (30, 5%); (35, 5%). The column oven and the autosampler temperatures were 40 and 15 °C, respectively, and the injection volume was 10 µL. The samples were prepared by dissolving 1 mg of dried extract in 1 mL of methanol and filtering the solutions through 0.22 µm PTFE syringe filters. All the solvents used were LC-MS grade from Carlo Erba (Val de Reuil Cedex, France).

MS parameters were optimized for each analyte individually, and the three transitions (or, in some cases, two) with the best signal–noise ratio were selected for the sMRM method development. The optimized sMRM parameters are compiled in Table S1 of Supplemental Material. ESI operation parameters were set as follows: source temperature 450 °C, curtain gas 35 psig, ion source gas 1 (GS1) 60 psig, ion source gas 2 (GS2) 60 psig, and collision set to high. The entrance potential (EP) and collision cell exit potential (CXP) were both −10 V in negative and 10 V in positive ionization modes. The ion capillary voltage was set to −4500 V in negative mode and 4500 V in positive mode.

For the quantification of phytochemicals, a calibration curve for each of them was made, with some exceptions: protocatechuic and syringic acids were quantified as 4-hydroxybenzoic acid; caffeic, chlorogenic and neochlorogenic acids were quantified as *p*-coumaric acid; apigenin and luteolin were quantified as apigenin-7-glucoside and luteolin-7-glucoside, respectively; quercetin, rutin and quercitrin were quantified as quercetin-3-glucoside. Due to the variability of the samples analyzed, it was necessary to prepare two different calibration ranges for the same analyte in some cases. The calibration curves’ determination coefficients were R^2^ > 0.994 in all cases.

### 5.4. Cells and Viruses

The host cells, African green monkey kidney cells (*Cercophitecus aethiops*, Vero cl 76 obtained from Laboratory of Cell Culture-Instituto de Virología “Dr. J. M. Vanella”, Argentina), were grown and maintained in a humid atmosphere at 37 °C with 5% CO_2_. The growth medium (GM) consists of Eagle’s minimal essential medium (E-MEM, Gibco, Grand Island, NY, USA) supplemented with 10% (*v*/*v*) fetal bovine serum (FBS, Natocor, Carlos Paz, Córdoba, Argentina), L-glutamine (30 μg/mL, Sigma-Aldrich, St. Louis, MO, USA), and gentamicin (50 μg/mL, Sigma-Aldrich). In contrast, the maintenance medium (MM) has the same formulation but with 2% FBS.

Herpes simplex virus type I (HSV-1) Kos strain (DNA virus) and chikungunya virus (CHIKV) ARG strain 2016 (RNA virus) were used. Virus stocks were propagated and titrated. The viral titration was expressed in plaque-forming units per milliliter (PFU/mL), 2 × 10^7^ PFU/mL for HSV-1 and 3 × 10^8^ PFU/mL for CHIKV.

### 5.5. Sample Solutions

Each extract was dissolved in dimethyl sulfoxide (DMSO) to obtain the stock solution (100 mg/mL), from which dilutions in MM were prepared to carry out the cytotoxicity and the antiviral activity assays. It is essential to explain that although the DMSO has demonstrated an antiviral effect on HSV-1 and cytotoxicity in different cell types, the final concentration used in our assays was consistently below 1%, a concentration that has no cytotoxic or antiviral effect [65,66].

### 5.6. In Vitro Cytotoxicity Assay

This assay determines the sample concentrations that do not affect the viability of the host cells to ensure that the observed cytopathic effect is due to the action of the virus and not to the toxicity of the extract. Employing the neutral red (NR) uptake assay, the cell viability (CV) was measured following a procedure previously described by other authors with some minor modifications [67]. A confluent monolayer of Vero cells (1 × 10^5^ cell/well ≈ 95% confluence), grown in 96-well culture plates, was exposed to 15 decreasing concentrations starting at 1 mg/mL of each extract, preparing three replicates of each concentration and including three wells of cells with MM as cell control (CC). After incubation for 72 h (37 °C in 5% CO_2_), the absorbance of the NR extracted from inside the cells was measured at 540 nm using a microplate reader (BioTek ELx800, Winooski, VT, USA). The percentage of CV (% CV) was calculated by comparing it with CC (100% CV). Using the Origin 8.6 software, each extract’s plot % CV vs. concentrations was graphed with non-linear regression analysis (Sigmoidal curve, R^2^ > 0.95).

From these graphs, the following toxicity parameters were estimated for each extract: the concentration that reduced the viable cells to 50% (CC_50_) and a subtoxic concentration (SubTC) defined as the concentration that causes 20% of cellular death and produces slight morphological changes observed by microscopy [68]. These values allowed us to use non-toxic or sub-toxic concentrations in antiviral activity assays.

### 5.7. Plaque Reduction Assay

#### 5.7.1. Antiviral Assays

The viral suspension (100 PFU/well) was inoculated into a confluent monolayer of Vero cells (4.8 × 10^7^ cells/well) grown in 24-well culture plates. After 1 h of adsorption at 37 °C, the residual inoculum was discarded, and the cells were washed with phosphate-buffered saline (PBS). Subsequently, the cells were covered with a semi-solid medium (1% agarose in a double concentration of E-MEM) and different extract concentrations (consecutive 2-fold serial dilutions from SubTC). Three replicates per concentration of each extract were assayed. Infected cells without extract, such as viral control (VC), uninfected cells without extracts as cell control (CC), and positive control were included in triplicate. Also, a DMSO control of 1% was added. According to the viral model, the PFU were counted after 3–5 days of incubation at 37 °C with 5% CO_2_, following the methodology described by Cheng et al. [69]. Results were expressed as a percentage of inhibition (%I) in correlation with VC, and %I was plotted according to the treatment concentration to calculate the effective concentration 50 (EC_50_). Inhibition percentages below 50% were considered extracts without activity [70].

The selectivity index (SI) indicates how suitable a sample or extract is for decreasing viral replication without damaging host cells. SI was measured as the ratio between the concentration that produces toxicity in 50% of the host cells (CC_50_) and the concentration that inhibits viral replication by 50% (EC_50_).

#### 5.7.2. Viral Inactivation Activity

The virucidal effect of each extract (capacity to inactivate the viral particles before they enter the host cell) was determined using the PFU reduction test in Vero cells. For this, 100 μL of the viral stock (see Section 2.4) was mixed with 100 μL of a concentration of each extract corresponding to its CC_50_ (1:1). Viruses in MM without extracts were used as viral control (VC), the CC_50_ of each extract in MM was included as extract control, and positive control was also tested. These mixtures were incubated for 1 h at 37 °C. Subsequently, serial dilutions (factor 10) were made from each mixture, and each dilution was added in duplicate to a confluent layer of Vero cells (4.8 × 10^7^ cells/well). After 1 h of incubation (37 °C in a humid atmosphere with 5% CO_2_), the monolayer was washed with PBS, and the cells were covered with a semi-solid medium (1% agarose in a double concentration of E-MEM). The PFU were counted after 3–5 days of incubation (37 °C with 5% CO_2_). The residual viral infectivity was determined by the PFU reduction assay [70]. Extracts that caused decreases ≤ 2 logarithms compared to the titer of the VC were considered extracts without virucidal activity [70].

### 5.8. Positive Controls

Acyclovir (Fada, CABA, Buenos Aires, Argentina) was used as a positive control of HSV-1 at a concentration of 15 μM. In contrast, a positive antiviral control against CHIKV was not included because of the lack of effective antiviral drugs for this virus.

### 5.9. Statistical Analysis

The CC_50_ and SubTC values were obtained from the dose–response curves generated from the non-linear regression analysis. Values were expressed as mean ± standard deviation ($\bar{x}\;$± SD) from 3 independent experiments. One-way analysis of variance (ANOVA) was performed for each type of extract to evaluate variability in cytotoxicity and antiviral activity among populations. To determine statistical significance, the post hoc Di Rienzo, Guzmán y Casanoves test (DGC) was applied [71] with a *p* ≤ 0.05. To assess variability among extract types (Aqu, CHCl_3_, EtOH) and populations (VP, TC, SG, PP), three multivariate analyses were performed: principal component analysis (PCA), hierarchical clustering analysis (HCA) combined with heatmap visualization, and partial least squares discriminant analysis (PLS-DA). PCA was conducted to evaluate the association between phytochemical compounds and *B. crispa* populations, focusing on their chemical profiles. PCA was based on a correlation matrix and performed using the FactoMineR and factoextra packages. To further assess clustering among populations, HCA and PLS-DA were applied. HCA, which utilized Euclidean distance matrix and Ward’s method for clustering, was visualized with a heatmap using the pheatmap package, while PLS-DA was conducted with the mixOmics package. Additionally, Pearson’s correlation was performed to explore potential associations between chemical profiles and antiviral activities against HSV-1 and CHIKV, using the corrplot package. All analyses were conducted in R Studio statistical software (version 4.2.1) [72,73,74,75,76,77].

## Figures and Tables

**Table 1:** Cytotoxic and antiviral activities of aqueous and organic extracts from four wild populations of *B. crispa.*

Extracts/ Positive Control	Population	CC_50_ (µg/mL) ^1^	SubTC (µg/mL) ^2^	CHIKV			HSV-1		
				I (%) ^3^	EC_50_ ^4^ (µg/mL)	SI ^5^	I (%)	EC_50_ (µg/mL)	SI
CHCl_3_	VP	326.2 ± 6.7 ^d^	134.9 ± 5.6 ^c^	45.7 ± 0.8 ^d^	-	-	17.6 ± 0.6	-	-
	TC	109.3 ± 4.6 ^b^	52.0 ± 2.6 ^a^	**59.4 ± 0.6 ^c^**	44.2 ± 0.2	2.5	41.3 ± 0.6	-	-
	SG	169.1 ± 5.7 ^c^	105.6 ± 10.1 ^b^	**67.0 ± 1.2 ^b^**	78.8 ± 0.5	2.1	48.3 ± 0.1	-	-
	PP	94.8 ± 1.6 ^a^	45.7 ± 0.4 ^a^	**81.6 ± 0.9 ^a^**	28.2 ± 0.1	3.4	**55.2 ± 0.4**	41.4 ± 0.1	2.3
EtOH	VP	92.9 ± 3.5 ^a^	20.2 ± 1.1 ^a^	35.9 ± 0.6 ^c^	-	-	32.5 ± 0.6	-	-
	TC	413.6 ± 9.5 ^c^	219.8 ± 5.4 ^c^	43.0 ± 0.9 ^c^	-	-	**57.6 ± 0.4 ^b^**	190.8 ± 0.3	2.2
	SG	159.2. ± 1.5 ^b^	103.6 ± 2.8 ^b^	**63.5 ± 0.9 ^b^**	81.6 ± 0.4	2	**61.6 ± 0.5 ^b^**	84.1 ± 0.6	1.9
	PP	571.9 ± 0.3 ^d^	326.8 ± 9.8 ^d^	**79.0 ± 0.8 ^a^**	188.7 ± 0.1	3	**78.9 ± 0.2 ^a^**	207.1 ± 0.1	2.8
Aqu	VP	700.4 ± 17.3 ^d^	482.0 ± 11.4 ^b^	27.8 ± 0.1 ^d^	-	-	19.4 ± 0.2	-	-
	TC	611.9 ± 9.1 ^c^	507.0 ± 0.7 ^c^	35.8 ± 0.2 ^c^	-	-	30.2 ± 0.2	-	-
	SG	422.3 ± 9.6 ^a^	342.8 ± 9.3 ^a^	**57.9 ± 1.1 ^b^**	296.0 ± 0.2	1.4	38.9 ± 0.1	-	-
	PP	573.4 ± 2.3 ^b^	491.9 ± 0.7 ^b^	**74.0 ± 0.6 ^a^**	332.4 ± 0.5	1.7	**51.2 ± 0.7**	480.4 ± 0.1	1.2
ACV	-	>200	-	-	-	-	100	>1000	>20,000

Data are mean values from three independent experiments $\bar{x}$ ± SD performed in triplicate. Values in bold font indicate positive extracts. CHCl_3_: chloroform extract; EtOH: ethanol extract; Aqu: aqueous extract; VP: Villa del Parque population; TC: Tala Cañada population; SG: San Geronimo population; PP: Puesto Pedernera population; CHIKV: chikungunya virus; HSV-1: herpes simplex virus type 1; ACV: acyclovir. ^1^ CC_50_: concentration that reduced the viable cells to 50%. ^2^ SubTC: concentration that ensures 80% of the viable cells. ^3^ Percentages of viral inhibition at SubTC of extracts of *B. crispa* on CHIKV or HSV-1. The results are expressed as a percentage of inhibition compared to viral controls (100%). ^4^ EC_50_: extract concentration that produces a 50% inhibition of the virus-induced cytopathic effect. ^5^ SI: The selectivity index was determined by the ratio of CC_50_/EC_50_. ^a–d^ Different letters indicate significant differences within the same type of extract between wild populations: *p* ≤ 0.05 (ANOVA DGC test).

## Data Availability

Data are contained within the article and Supplementary Materials.

## Supplementary Materials

The following supporting information can be downloaded at: https://www.mdpi.com/article/10.3390/plants13213077/s1. Table S1: Retention time (Rt) and MRM parameters: optimized collision energy (CE), declustering potential (DP), and selected transitions for each phytochemical. Table S2: Phytochemical analysis of aqueous and organic extracts from four wild populations of B. crispa. Results are expressed as mean (mg/g extract) ± SD (n = 3), except when the quantification was impossible because values were below the quantification limit (*). Nd, not detected. Figure S1: Curves of cellular viability percentage vs. concentration of the ethanolic (EtOH), chloroform (CHCl3), and aqueous (Aqu) extracts for the four populations of B. crispa on Vero cells, evaluated by NR uptake assay. Each result is expressed as mean ± SD of three independent experiments performed in triplicate. A-Villa del Parque Population (VP); B-Tala Cañada Population (TC); C-San Gerónimo Population (SG); D-Puesto Pedernera Population (PP). Figure S2: Curves of the percentage of inhibition of each viral model vs. the concentration of the ethanolic (EtOH), chloroform (CHCl3), and aqueous (Aqu) extracts of the four populations of B. crispa. Each result is expressed as mean ± SD of three independent experiments performed in triplicate. A-Villa del Parque Population (VP); B-Tala Cañada Population (TC); C-San Gerónimo Population (SG); D-Puesto Pedernera Population (PP).
