# Supplementary material for: Antiviral Potential and Chemical Composition of Wild Baccharis crispa Spreng. Populations (Asteraceae) from Córdoba, Argentina: Perspective on Population Variability
Source: Plants (Basel). 2024 Nov 1;13(21):3077. doi: 10.3390/plants13213077 (PMC11548641; doi:10.3390/plants13213077)

Legends of Supplemental File

Table S1: Retention time (Rt) and MRM parameters: optimized collision energy (CE), declustering potential (DP), and selected transitions for each phytochemical.

Table S2: Phytochemical analysis of aqueous and organic extracts from four wild populations of *B. crispa*. Results are expressed as mean (mg/g extract) ± SD (n = 3), except when the quantification was impossible because values were below the quantification limit (*). Nd, not detected.

Figure S1: Curves of cellular viability percentage *vs.* concentration of the ethanolic (EtOH), chloroform ($CHCl_3$), and aqueous (Aqu) extracts for the four populations of *B. crispa* on Vero cells, evaluated by NR uptake assay. Each result is expressed as mean ± SD of three independent experiments performed in triplicate. A-Villa del Parque Population (VP); B-Tala Cañada Population (TC); C-San Gerónimo Population (SG); D-Puesto Pedernera Population (PP).

Figure S2: Curves of the percentage of inhibition of each viral model *vs.* the concentration of the ethanolic (EtOH), chloroform ($CHCl_3$), and aqueous (Aqu) extracts of the four populations of *B. crispa*. Each result is expressed as mean ± SD of three independent experiments performed in triplicate. A-Villa del Parque Population (VP); B-Tala Cañada Population (TC); C-San Gerónimo Population (SG); D-Puesto Pedernera Population (PP).

Table S1

Analyte	Rt (min)	Precursor ion (*m/z*)	Product ions (*m/z*)	Declustering Potential (V)	Collision Energy (V)	Identified in *B. crispa extracts*
**Hydroxycinnamic acids & derivatives**						
Caffeic acid	8.9	179.0	135 / 134 / 89	-20	-20 / -30 / -40	+
Caftaric acid	6.2	311.0	149 / 135 / 179	-20	-20 / -40 / -20	-
Chicoric acid	10.7	472.5	311 / 149 / 179	-20	-20 / -30 / -40	-
3-O-caffeoylquinic acid	9.9	353.0	191 / 85 / 127	-20	-40 / -50 / -50	+
5-O-caffeoylquinic acid	6.7	353.0	191 / 135 / 179	-20	-40 / -50 / -40	+
Cinnamic acid	17.2	147.0	103 / 77	-60	-20 / -30	-
p-Coumaric acid	10.3	163.0	119 / 93 / 117.5	-40	-20 / -40 / -50	+
trans-Coutaric acid	8.3	295.0	163 / 119 / 149	-20	-30 / -30 -30	-
Ferulic acid	11.5	193.0	134 / 178 / 149	-60	-30 / -20 / -20	+
Sinapic acid	12.2	223.0	208 / 164 / 149	-20	-20 / -20 / -30	+
Verbascoside	11.4	623.0	161 / 133 / 315	-40	-40 / -100 / -40	+
**Hydroxybenzoic acids & derivatives**						
Ellagic acid	12.1	301.0	145 / 185 / 229	-60	-50 / -40 / -40	-
Gallic acid	2.1	169.0	125 / 97 / 80	-40	-20 / -30 / -40	+
Gentisic acid	6.16	153.0	108 / 81	-40	-30 / -20	+
4-Hydroxybenzoic acid	7.12	137.0	93 / 65 / 75	-20	-20 / -40 / -40	+
Protocatechuic acid	4.18	153.0	109 / 108 / 123	-60	-20 / -40 / -20	+
Syringic acid	10.2	197.0	182 / 123 / 95	-40	-20 / -30 / -40	+
Vanillic acid	10.3	167.0	152 / 124 / 108.5	-40	-20 / -20 / -20	-
Methyl gallate	8.2	183.0	124 / 78 / 168	-80	-30 / -40 / -20	-
**Flavonoids**						
<u>**Flavanols**</u>						
(+)-Catechin	8.1	289.0	245 / 123 / 109	-80	-20 / -40 / -20	-
Catechin 3-O-gallate	10.7	441.0	169 / 125 / 289	-80	-30 / -60 / -30	-
(-)-Epicatechin	9.6	289.0	245 / 123 / 109	-80	-20 / -40 / -50	-
Epicatechin 3-O-gallate	10.6	441.0	169 / 125 / 289	-80	-30 / -50 /-30	-

(-)-Epigallocatechin	7.8	305.0	125 / 179 / 138	-20	-30 / -20 / -40	-
(-)-Epigallocatechin gallate	9.7	457.0	169 / 125 / 305	-60	-50 / -30 / -30	-
Procyanidin A2	11.1	577.0	286.5 / 424.5 / 137.5	60	40 / 20 / 80	-
Procyanidin B1	9.4	578.0	289 / 409 / 275	20	30 /30 / 30	-
Procyanidin B2	9.4	579.0	291 / 409 / 426.5	60	20 / 30 / 20	-
Theaflavin	18.4	563.0	241 / 269 / 137	-40	-50 / -50 / -60	-
**Flavonols**						
Fisetin	13.3	285.0	135 / 121 / 91	-100	-30 / -30 / -40	-
Isorhamnetin	20.8	315.0	300 / 151 / 107	-80	-30 / -40 / -50	+
Kaempferide	27.7	299.0	284 / 151 / 107	-20	-30 / -40 / -50	-
Kaempferol	18.6	285.0	185 / 159 / 151	-60	-40 / -50 / -40	+
Morin	13.7	301.0	151 / 125 /107	-60	-30 / -30 - /40	-
Myricetin	12.7	317.0	179 / 151 / 137	-40	-330 / -30 / -40	-
Quercetin	15.7	301.0	151 / 179 / 121	-80	-30 / -30 /-50	+
Quercetin 3-glucoside	11.6	463.0	300 / 271 / 179	-20	-50 / -40 /-40	+
Quercetin 3-glucuronide	11.5	477.0	301 / 179 / 151	-60	-40 / -50 / -40	+
Quercetin 3-galactoside	11.6	463.0	300 / 271 / 255	-60	-40 / -50 / -50	+
Quercetin 3,4'-di-O-glucoside	10.5	625.0	463 / 301 / 179	-40	-30 / -50 / -50	-
Quercitrin	12.8	447.0	300 / 271 / 151	-40	-30 / -50 / -50	+
Rutin	11.4	609.0	300 / 271 / 151	-40	-50 / -40 / -50	+
**Flavones**						
Apigenin	19.6	269.0	117 / 151 / 107	-40 / -80 / -80	-38 / -30 / -40	+
Apigenin 7-glucoside	14.1	431.0	268 / 211 / 239	-140	-50 / -80 / -60	+
Diosmin	16.5	607.0	299 / 284 / 256	-80	-30 / -60 / -100	-
Luteolin	16.8	285.0	133 / 151 / 175	-80	-40 / -50 / -30	+
Luteolin 7-glucoside	12.1	447.0	285 / 133 / 151	-140	-40 / -80 / -60	+
Tangeretin	28.5	373.0	183 / 299 / 327	60	60 / 50 / 50	-
**Flavanones**						
Eriodictyol	15.4	287.0	151 / 135 / 107	-80	-20 / -40 / -30	+
Hesperetin	22.2	301.0	164 / 136 / 151.5	-40	-40 / -40 / 50	-
Hesperidin	16.5	609.0	301 / 164 / 286	-80	-50 / -60 / -50	-
Naringenin	18.8	271.0	151 / 119 / 107	-40	-30 / -30 / -30	+
Naringin	14.4	579.0	271 / 151 / 175	-80	-40 / -50 / -40	-
Narirutin	13.9	579.0	271 / 151 / 119	-40	-30 / -60 - /80	-

Taxifolin	10.8	303.0	285 / 125 / 151	-80	-20 / -30 / -30	+
**Isoflavonoids**						
Genistein	19.3	269.0	133 / 159 /63	-80	-40 / -40 / -60	+
**Anthocyanins**						
Cyanidin	10.7	287.0	241 / 137 / 213	30	40 / 40 / 40	-
Cyanidin 3-glucoside	9.6	449.0	287 / 213 / 241	80	40 / 80 / 80	-
Cyanidin 3-O-rutinoside	9.8	595.0	287 / 449 / 213	60	50 / 30 / 50	-
Cyanidin 3,5-O-diglucoside	8.5	611.0	287 / 449 / 213	20	50 / 30 / 120	-
Delphinidin	15.7	303.0	229 / 257 / 202	40	50 / 50 / 40	-
Delphinidin 3-O-glucoside	11.6	465.0	303 / 229 / 257	40	40 / 70 / 50	+
Malvidin	10.2	331.0	315 / 287 / 241.5	30	40 / 50 / 50	-
Malvidin 3-O-glucoside	10.3	493.0	331 / 316 / 287.5	20	30 / 80 / 80	-
Malvidin 3,5-O-diglucoside	8.5	655.0	331 / 492 / 315	40	50 / 30 / 80	-
Pelargonidin	12.8	271.0	121 / 198 / 93	80	50 / 40 / 60	-
Pelargonidin 3-O-glucoside	10.1	433.0	272 / 141 /169	40	40 / 80 / 80	-
Peonidin	21.6	301.0	287 / 259	40	40 / 40	-
Peonidin 3-O-glucoside	10.3	462.0	201 / 229 / 257	100	80 / 80 / 60	-
Petunidin	11.3	317.0	246 / 203 / 274	60	50 / 50 / 40	-
Petunidin 3-O-glucoside	9.7	479.0	317 / 303 / 217	20	30 / 60 / 80	-
**Dihydrochalcones**						
Phloretin	17.0	273	123 / 81	-40	-30 / -40	-
Phloridzin	12.6	435	273 / 167 / 123	-60	-20 / -40 / -50	-
**Stilbenes**						
Astringin	11.0	405	243 / 159 / 201	-40	-30 / -60 / -50	-
Piceatannol	12.0	243	201 / 159 / 175	-80	-30 / -30 / -30	-
Polydatin	12.7	389	227 / 185 / 143	-80	-30 / -50 / -50	-
trans-Resveratrol	14.4	227	143 / 185 / 119	-20	-30 / -20 / -40	-
ε-Viniferin	16.6	453	347 / 225 / 198	-80	-40 / -50 / -50	-
**Lignans**						
Enterolactone	20.1	297	253 / 107 / 189	-80	-30 / -40 / -30	-
Hinokinin	28.3	353	336 / 96 / 318	40	20 / 40 / 30	-

Magnolin	27.9	417	279 / 327 / 380	20	10 / 20 / 20	-
Pinoresinol	19.2	357	151 / 136	-80	-20 / -30	+
Secoisolariciresinol	13.9	361	165 / 121 / 179	-80	-30 / -50 / 40	+
**Coumarins**						
Coumarin	16.0	147	103 / 91 / 65	40	20 / 40 / 50	+
Esculetin	9.7	177	105 / 89 / 133	-60	-30 / -30 / -20	+
Scopoletin	12.8	191	176 / 104 / 148	-40	-20 / -30 / -30	+
Umbelliferone	11.9	161	133 / 105 / 77	-80	-30 / -30 / -40	+
**Other phenolic compounds**						
Arbutin	1.2	271	108 / 161 / 71	-80	-10 / -50 / -20	-
Coniferyl alcohol	10.5	179	146 / 135 / 165	-80	-20 / -20 / -20	-
Hydroxytyrosol	5.0	153	123 / 95 / 93	-80	-20 / -30 / -30	+
Resorcinol	3.7	111	66 / 94 / 84	60	30 / 20 / 20	-
Pyrogallol	2.5	125	79 / 97 / 107	-60	-30 / -20 / -20	+
**Terpenes**						
11β,13-Dihydrolactucopicrin	24.7	412	261 / 187 / 215	80	20 / 40 / 30	-
Lactucin	10.9	277	213 / 142 / 129	80	20 / 40 / 50	-
Lactucopicrin	24.8	409	257 / 213 / 151	-80	-20 / -30 / -20	-

Table S2

Phytochemical Family	Compound	Wild population											
		Puesto Pedernera			Villa del Paque			Tala Cañada			San Geronimo		
		EtOH	$CHCl_3$	Aqu	EtOH	$CHCl_3$	Aqu	EtOH	$CHCl_3$	Aqu	EtOH	$CHCl_3$	Aqu
Hydroxybenzoic Acids	4-Hydroxybenzoic acid	42,38 ± 1,75	7,44 ± 0,64	54,59 ± 12,03	56,33 ± 4,26	6,62 ± 2,68	37,88 ± 1,32	52,10 ±10,54	7,37 ±0,82	137,57 ±19,96	51,45 ±1,01	8,32 ± 0,57	27,3 ± 1,28
	Gallic acid	*	*	10,67 ± 4,39	*	*	*	*	*	7,85 ± 1,88	*	*	*
	Protocatechuic ACID*	433,11 ± 43,65	2,3 ± 0,34	1217,37 ± 485,8	277,82 ± 8,12	2,27 ± 2,32	185,09 ± 15,02	64,84 ± 3,66	0,40 ± 0,11	400,88 ± 60,06	87,15 ± 4,27	0,65 ± 0,11	50,97 ± 1,58
	Syringic acid*	6,17 ± 0,73	2,88 ± 0,37	3,09 ± 0,06	2,1 ± 0,3	1,21 ± 0,09	2,16 ± 0,07	3,8 ± 0,44	1,77 ± 0,08	395,10 ± 58,28	4,08 ± 0,19	0,6 ± 0,21	1,11 ± 0,17
	Gentisic acid	4,08 ± 0,34	*	27,04 ± 3,78	3,21 ± 0,56	*	44,05 ± 2,34	6,41 ± 0,29	*	667,26 ± 46,5	4,2 ± 0,66	*	4,01 ± 0,47
Hydroxycinnamic Acids	*p*-coumaric acid	16,75 ± 1,08	17,15 ± 3,15	5,36 ± 0,86	15,92 2,44	2,56 ± 0,33	21,24 ± 1,96	17,23 ± 4,18	5,28 ± 0,38	7,74 ± 0,62	12,91 ± 1,2	6,05 ± 0,63	9,66 ± 1,29
	Caffeic acid*	161,3 ± 17,9	*	134,86 ± 18,42	141,3 ± 6,23	*	680,97 ± 28,93	121,22 ± 8,27	*	75,46 ± 2,17	95,29 ± 2,29	*	108,59 ± 2,9
	3-O-caffeoylquinic acid	642,62 ± 304,5	*	890,57 ± 12,3	290,5 ± 10,72	*	883,7 ± 117,28	565,52 ± 138,56	*	29,87 ± 9,86	454,65 ± 152,35	*	924,67 ± 163,12
	5-O-caffeoylquinic acid	195,38 ± 35,54	nd	197,34 ± 29,37	71,68 ± 11,86	*	127,8 ± 19,81	63,00 ± 4,88	nd	*	73,48 ± 17,05	*	90,13 ± 9,21
	Ferulic acid	17,2 ± 1,3	40,20 ± 4,2	52,05 ± 5,49	27,27 ± 5,09	24,39 ± 0,93	41,32 ± 2,69	22,47 ± 1,8	29,69 ± 0,65	87,39 ± 13,25	23,67 ± 2,24	25,52 ± 4,16	16,17 ± 1,37
	Sinapic acid	1,06 ± 0,15	0,86 ± 0,07	nd	1,13 ± 0,1	0,78 ± 0,16	*	0,75 ± 0,02	nd	nd	1,17 ± 0,14	0,77 ± 0,09	nd
	Verbascoside	nd	*	nd	nd	nd	nd	nd	nd	14,18 ± 2,66	nd	nd	nd
Flavone	Apigenin-7-glucoside	7,44 ± 1,52	nd	*	69,24 ± 4,08	nd	15,12 ± 1,25	67,11 ± 3,03	nd	nd	95,58 ± 8,55	nd	19,17 ± 1,07
	Apigenin*	2544,26 ± 248,09	2124,23 ± 614,08	1238,50 ± 133,5	7231,16 ± 1133,78	*	*	5638,75 ± 799,04	*	*	6820,11 ± 122,1	4994,45 ± 664,45	*
	Luteonin-7-glucoside	5,8 ± 0,74	*	4,15 ± 1,75	48,10 ± 2,06	*	14,46 ± 2,87	19,75 ± 0,46	*	*	89,52 ± 17,39	*	19,39 ± 4,48
	Luteonin*	1302,63 ± 128,29	161,02 ± 29,53	156,67 ± 36,08	1756,49 ± 269,75	46,9 ± 6,19	92,64 ± 5,61	821,29 ± 183,94	28,43 ± 2,37	31,56 ± 12,04	2973,68 ± 231,49	466,02 ± 47,98	120,84 ± 1,02
Isoflavone	Genistein	6,04 ± 0,75	7,16 ± 1,09	*	11,08 ± 1,24	3,51 ± 0,5	*	14,37 ± 0,49	2,88 ± 0,15	*	12,14 ± 1,23	14,65 ± 1,65	*
Flavanone	Eriodictyol	8,54 ± 0,77	3,21 ± 0,33	1,12 ± 0,17	34,47 ± 2,1	2,10 ± 0,12	1,79 ± 0,05	8,07 ± 1,24	0,77 ± 0,04	2,91 ± 1,32	8,11 ± 0,57	5,05 ± 0,43	0,87 ± 0,21
	Naringenin	10,52 ± 0,06	41,5 ± 1,97	*	99,09 ± 12,49	60,55 ± 3,12	*	13,28 ± 0,85	17,22 ± 1,12	*	*	70,40 ± 3,77	*
Flavanonol	Taxifolin	*	*	nd	*	nd	*	nd	nd	4,07 ± 0,86	nd	*	nd
Flavonol	Quercetin 3-glucoside	66,71 ± 18,77	nd	*	49,27 ± 12,08	nd	21,64 ± 1,38	45,56 ± 8,03	nd	*	60,0 ± 6,49	nd	37,38 ± 6,33
	Quercetin*	23,76 ± 1,76	*	120,32 ± 23,65	*	*	28,84 ± 2,63	*	*	*	20,08 ± 0,25	*	*
	Rutin*	540,85 ± 42,12	*	*	145,58 ± 19,87	*	117,39 ± 8,36	191,12 ± 22,63	*	*	123,7 ± 16,29	*	143,85 ± 15,95
	Quercitrin	1,9 ± 0,22	nd	10,17 ± 1,55	9,16 ± 0,84	*	1,71 ± 0,07	6,46 ± 0,46	*	11,61 ± 2,5	12,67 ± 0,89	nd	4,17 ± 0,41
	Quercetin 3-glucuronide	8,94 ± 0,7	nd	8,93 ± 1,78	4,97 ± 0,4	nd	14,71 ± 0,63	22,17 ± 32,32	nd	nd	16,49 ± 0,81	nd	13,34 ± 0,77
	Quercetin 3-galactoside	362,28 ± 97,83	*	99,98 ± 5,98	274,44 ± 2,54	*	133,31 ± 2,4	249,56 ± 41,66	*	*	317,51 ± 29,06	*	216,3 ± 29,01
	Kaempferol	36,48 ± 1,94	9,25 ± 1,46	27,27 ± 2,72	78,98 ± 9,08	7,55 ± 0,63	12,81 ± 1,71	70,45 ± 6,15	10,5 ± 1,17	*	45,21 ± 4,04	41,19 ± 2,55	3,78 ± 0,54
	Isorhamnetin	19,97 ± 6,63	11,81 ± 3,1	63,91 ± 14,91	17,04 ± 2,03	14,04 ± 1,12	7,32 ± 1,1	17,05 ± 1,87	10,37 ± 1,16	3,79 ± 1,41	14,34 ± 0,31	20,36 ± 0,94	nd
Anthocyanin	Delphinidin	86,02 ± 8,97	*	371,55 ± 118,68	*	nd	nd	*	nd	98,32 ± 11,38	70,3 ± 9,33	*	*
	Delphinidin 3-O-glucoside	649,78 ± 121,6	nd	98,88 ± 32,51	350,37 ± 54,86	*	126,29 ± 25,41	307,82 ± 53,1	nd	2,15 ± 0,24	344,52 ± 62,79	nd	186,54 ± 44,65
Liganan	Pinoresinol	nd	15,4 ± 1,18	20,58 ± 0,88	6,52 ± 1,27	31,55 ± 3,82	9,69 ± 0,13	11,94 ± 1,12	48,85 ± 9,02	45,94 ± 18,12	nd	32,68 ± 2,45	5,03 ± 0,71
	Secoisolariciresinol	5,93 ± 0,73	13,49 ± 0,17	30,09 ± 7,15	5,83 ± 0,49	34,93 ± 3,41	69,15 ± 3,08	3,11 ± 0,24	30,87 ± 2,77	73,25 ± 18,73	1,82 ± 0,24	17,99 ± 3,14	15,85 ± 2,8
Hydroxycoumarin	Esculetin	10,15 ± 1,33	*	20,27 ± 4,53	*	*	17,09 ± 3,25	*	*	47,07 ± 13,1	*	*	11,04 ± 1,13
	Scopoletin	*	0,97 ± 0,08	1,6 ± 0,23	*	1,44 ± 0,12	2,04 ± 0,1	*	*	10,4 ± 1,44	*	*	*
	Coumarin	*	*	*	*	*	*	*	*	0,47 ± 0,03	*	*	*
	Umbelliferone	123,18 ± 23,27	*	73,34 ± 11,98	118,95 ± 14,92	nd	83,62 ± 5,23	115,77 ± 9,11	nd	3,77 ± 1,08	101,18 ± 8,81	nd	70,16 ± 10,67
Others	Hydroxytyrosol	nd	nd	11,72 ± 2,85	nd	nd	6,65 ± 0,45	nd	nd	98,74 ± 3,65	nd	nd	2,63 ± 0,33
	Pyrogallol	nd	nd	47,4 ± 5,77	nd	nd	nd	nd	nd	nd	nd	nd	nd

Figure S1

A-Villa del Parque Population

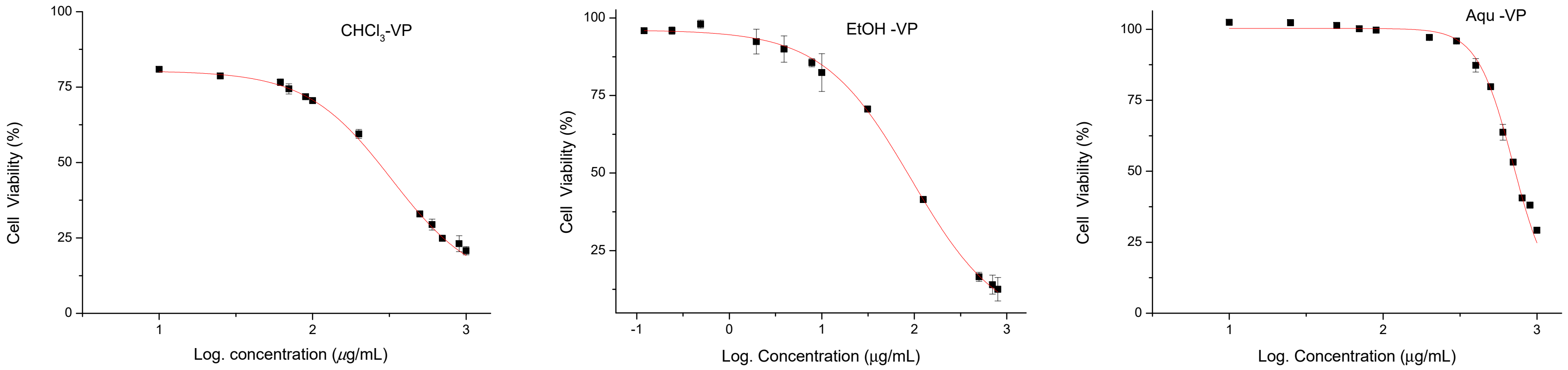

B- Tala Cañada Population

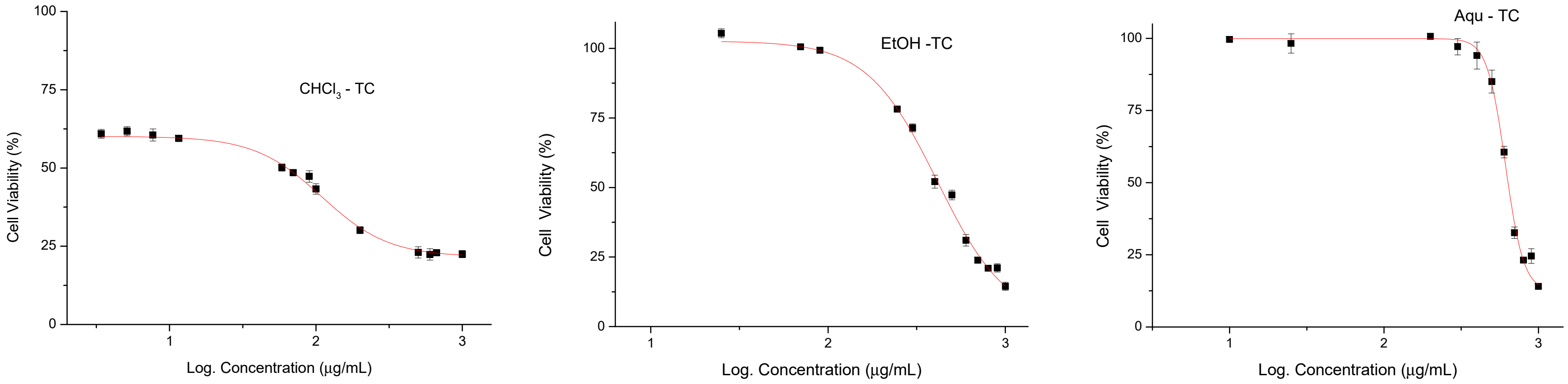

C- San Gerónimo Population

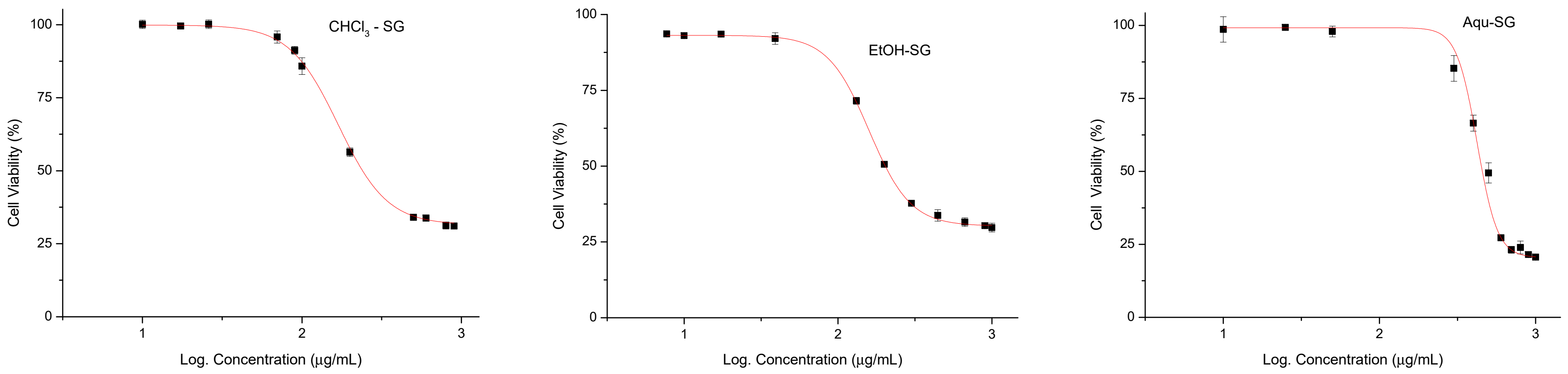

D- Puesto Pedernera Population

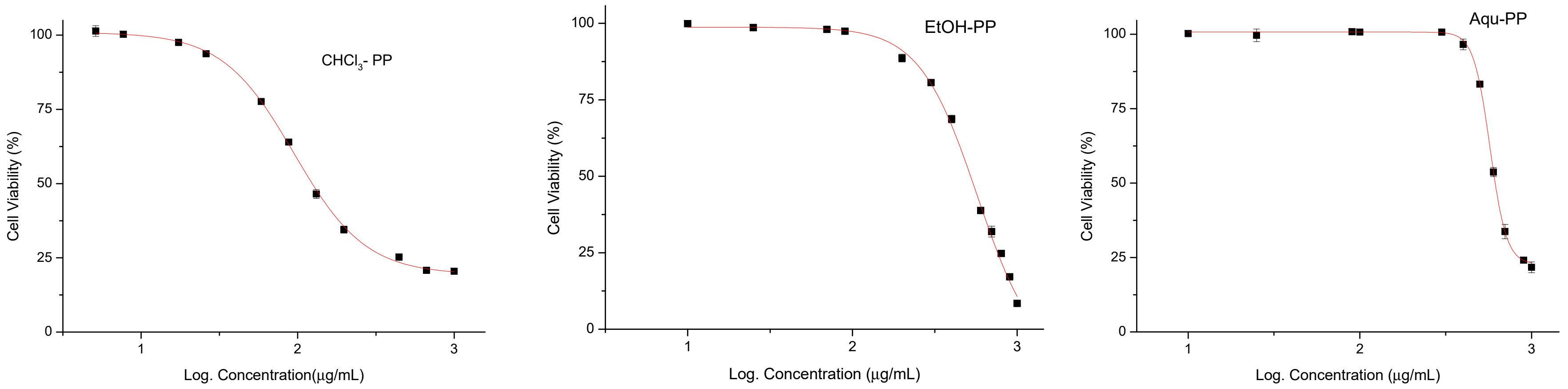

Figure S2

A-Villa del Parque Population

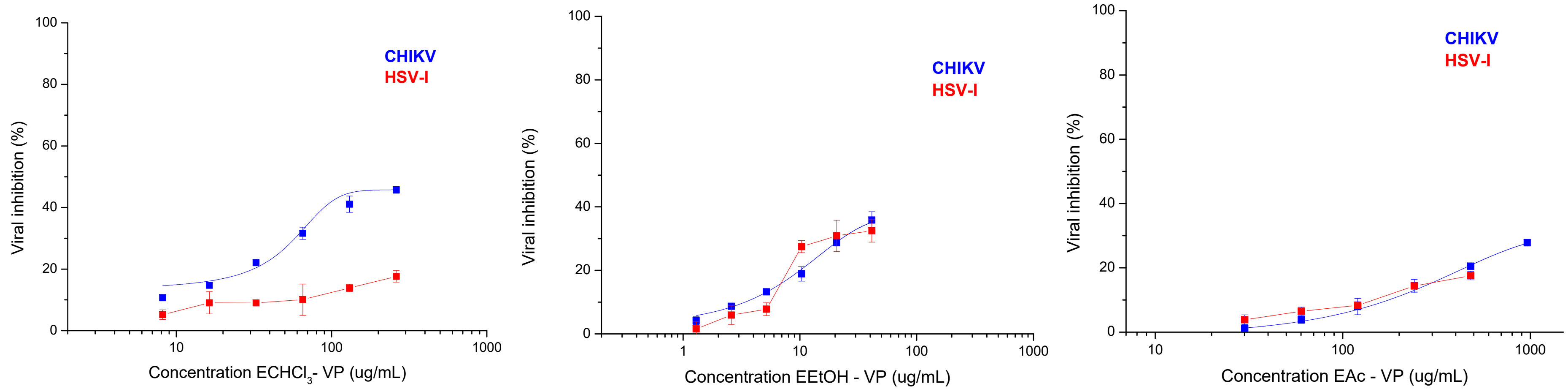

B-Tala Cañada Population

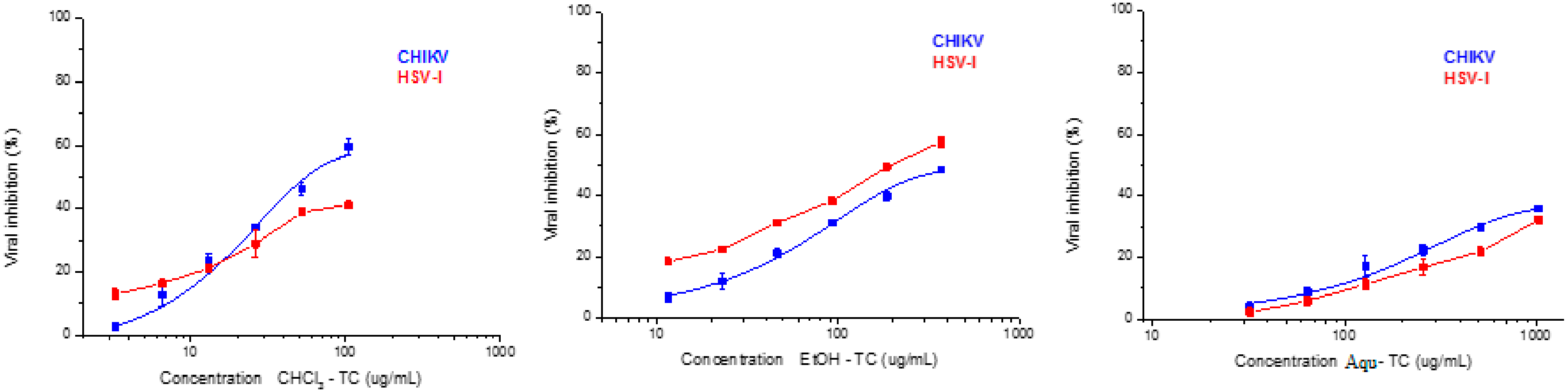

C-San Gerónimo Population

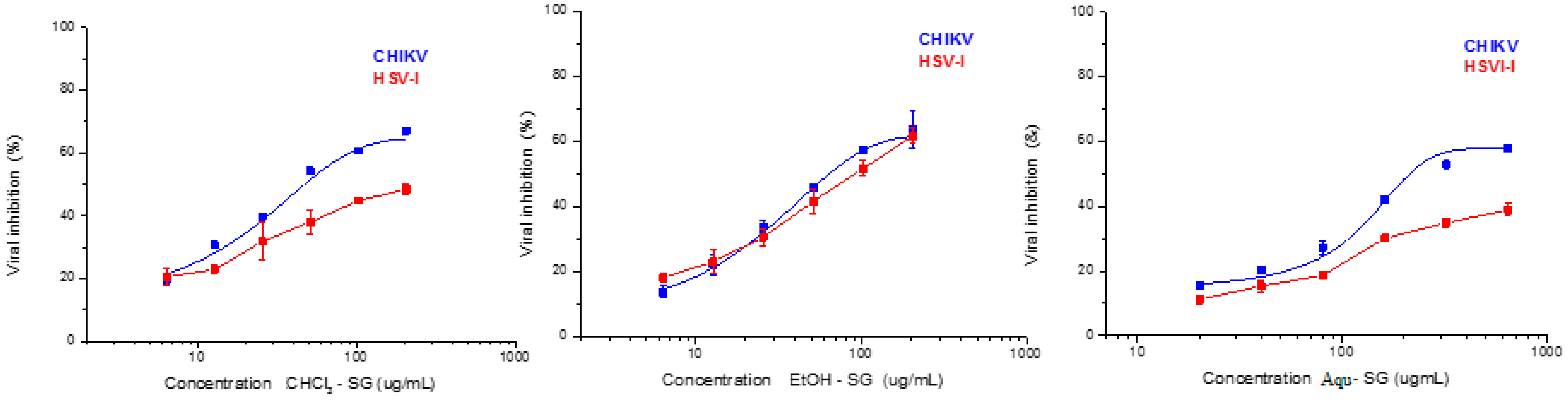

D-Puesto Pedernera Population

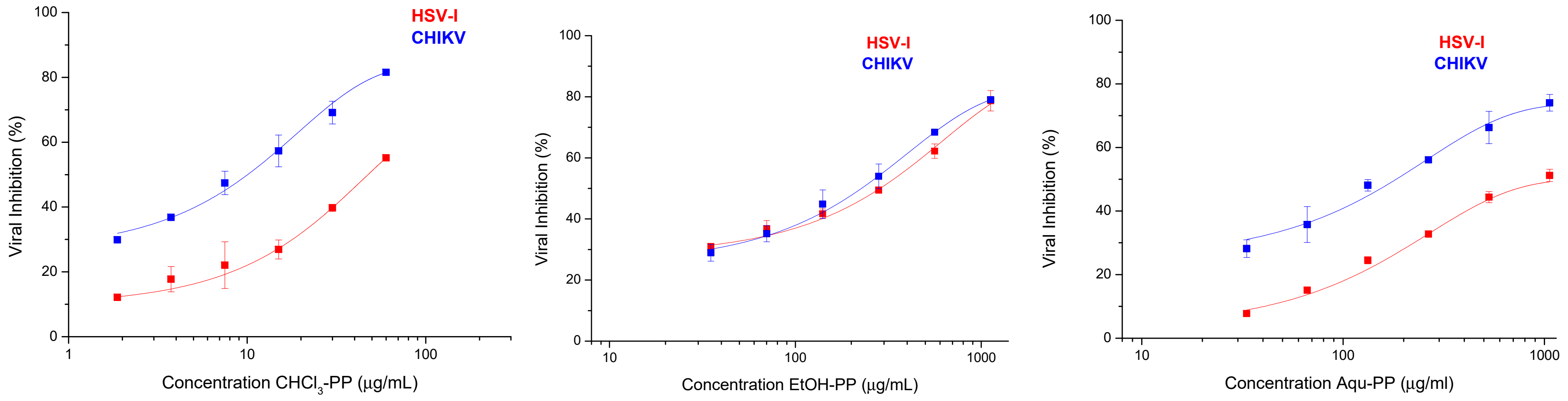

Supplement: Supplementary file 1 [file plants-13-03077-s001.zip › plants-3262910-supplementary.pdf]
